# (3,5-Dimethyl­phen­yl)[8-(3,5-dimethyl­benzo­yl)-2,7-dimeth­oxy­naphthalen-1-yl]methanone

**DOI:** 10.1107/S1600536812012202

**Published:** 2012-03-28

**Authors:** Toyokazu Muto, Kosuke Sasagawa, Akiko Okamoto, Hideaki Oike, Noriyuki Yonezawa

**Affiliations:** a Department of Organic and Polymer Materials Chemistry, Tokyo University of Agriculture & Technology, 2-24-16 Naka-machi, Koganei, Tokyo 184-8588, Japan

## Abstract

In the title mol­ecule, C_30_H_28_O_4_, the inter­planar angle between the two benzene rings of the 3,5-dimethyl­benzoyl groups is 50.35 (7)°. The dihedral angles between the two benzene rings and the naphthalene ring system are 81.87 (6) and 83.55 (6)°. In addition, the conformations of the pairs of methyl groups and their counterparts differ from each other though their environment is very similar. In the crystal, weak C—H⋯O inter­actions occur.

## Related literature

For electrophilic aromatic substitution of naphthalene deriv­atives, see: Okamoto & Yonezawa (2009 ▶); Okamoto *et al.* (2011 ▶). For the structures of closely related compounds, see: Muto *et al.* (2010 ▶, 2011*a* ▶,*b* ▶; 2012 ▶).
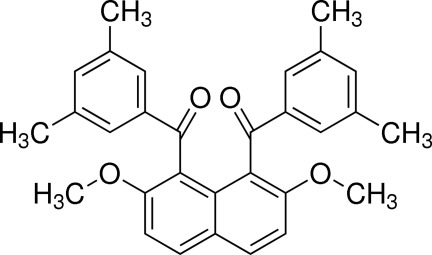


## Experimental

### 

#### Crystal data

C_30_H_28_O_4_
*M* *_r_* = 452.52
Monoclinic, 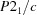

*a* = 19.4659 (3) Å
*b* = 8.27808 (10) Å
*c* = 15.8244 (2) Å
β = 110.69°
*V* = 2385.46 (6) Å^3^
*Z* = 4
Cu *K*α radiation
μ = 0.66 mm^−1^
*T* = 193 K
0.50 × 0.20 × 0.10 mm

#### Data collection

Rigaku R-AXIS RAPID diffractometer
Absorption correction: numerical (*NUMABS*; Higashi, 1999 ▶) *T*_min_ = 0.734, *T*_max_ = 0.937
43008 measured reflections
4360 independent reflections
3884 reflections with *I* > 2σ(*I*)
*R*_int_ = 0.032

#### Refinement

*R*[*F* ^2^ > 2σ(*F* ^2^)] = 0.038
*wR*(*F* ^2^) = 0.118
*S* = 1.08
4360 reflections
314 parameters
H-atom parameters constrained
Δρ_max_ = 0.22 e Å^−3^
Δρ_min_ = −0.16 e Å^−3^

### 

Data collection: *PROCESS-AUTO* (Rigaku, 1998 ▶); cell refinement: *PROCESS-AUTO*; data reduction: *CrystalStructure* (Rigaku/MSC, 2004 ▶); program(s) used to solve structure: *SIR2004* (Burla *et al.*, 2005 ▶); program(s) used to refine structure: *SHELXL97* (Sheldrick, 2008 ▶); molecular graphics: *ORTEPIII* (Burnett & Johnson, 1996 ▶); software used to prepare material for publication: *SHELXL97*.

## Supplementary Material

Crystal structure: contains datablock(s) I, global. DOI: 10.1107/S1600536812012202/fb2243sup1.cif

Structure factors: contains datablock(s) I. DOI: 10.1107/S1600536812012202/fb2243Isup2.hkl

Supplementary material file. DOI: 10.1107/S1600536812012202/fb2243Isup3.cml

Additional supplementary materials:  crystallographic information; 3D view; checkCIF report

## Figures and Tables

**Table 1:** Hydrogen-bond geometry (Å, °)

*D*—H⋯*A*	*D*—H	H⋯*A*	*D*⋯*A*	*D*—H⋯*A*
C7—H7⋯O1^i^	0.95	2.55	3.1332 (17)	120
C25—H25*B*⋯O2^ii^	0.98	2.41	3.170 (2)	134
C26—H26*A*⋯O1^i^	0.98	2.59	3.475 (2)	150

Symmetry codes: (i) 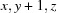; (ii) 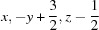.

**Fig. 1.**
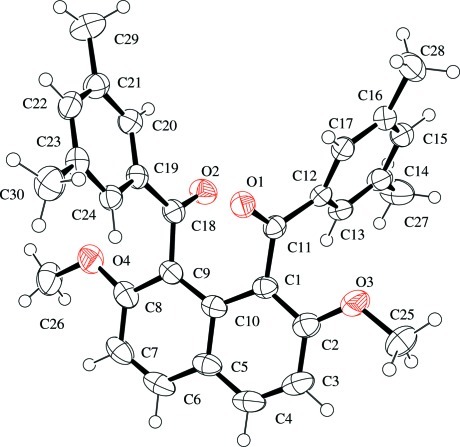
The title molecule with the displacement ellipsoids drawn at the 50% probability level.

**Fig. 2.**
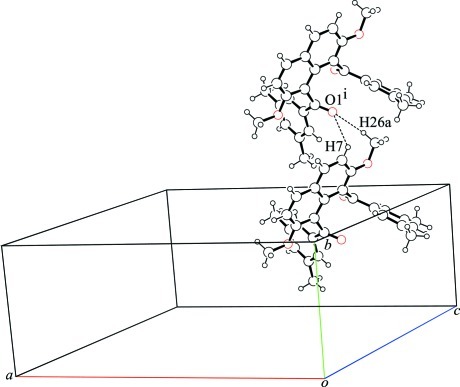
Two weak intermolecular C—H···O interactions [distances: H7···O1i = 2.55 Å and H26a···O1i = 2.59 Å; symmetry code: (i) x, y + 1, z].

**Fig. 3.**
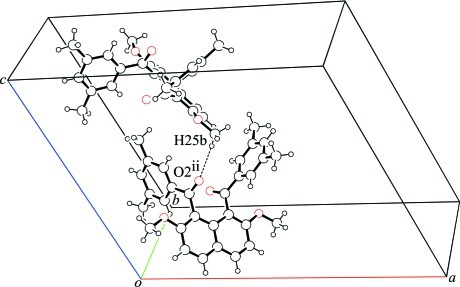
A week intermolecular C25—H25b···O2ii interaction [distance: H25b···O2 = 2.41 Å; symmetry code: (ii) x, -y + 3/2, z - 1/2].

## supplementary crystallographic information

### Comment

In the course of our study on electrophilic aromatic aroylation of 2,7-dimethoxynaphthalene, *peri*-aroylnaphthalene compounds have proven to be formed regioselectively with the aid of suitable acidic mediators (Okamoto & Yonezawa, 2009; Okamoto, Mitsui *et al.*, 2011). We have recently reported crystal structures of several 1,8-diaroylated naphthalene analogues exemplified by 1,8-bis(4-methylbenzoyl)-2,7-dimethoxynaphthalene (Muto *et al.*, 2010) and 1,8-bis(2,4,6-trimethylbenzoyl)-2,7-dimethoxynaphthalene (Muto *et al.*, 2012). In these compounds, the aroyl groups at the 1,8-positions of the naphthalene rings contain almost 90°. In addition, crystal structures of 1-monoaroylated naphthalene derivatives and the *β*-isomers of 3-monoaroylated derivatives have been also determined such as (2,7-dimethoxynaphthalen-1-yl)(2,4,6-trimethylphenyl)methanone (Muto *et al.*, 2011*a*) and (3,6-dimethoxynaphthalen-2-yl)(2,4,6-trimethylphenyl)methanone (Muto *et al.*, 2011*b*).

As a part of our continuing study on the molecular structures of these homologous molecules, the crystal structure of title compound, *peri*-aroylnaphthalene bearing two methyl groups at 3,5-positions on the phenyl group, is discussed in this article.

The title molecule is displayed in Fig. 1. Two 3,5-dimethylphenyl groups are out of the plane of the naphthalene ring. The interplanar angle between the best planes of the two phenyl rings (C12\C17 and C19\C24) is 50.35 (7)°. On the other hand, the two interplanar angles between the best planes of the 3,5-dimethylphenyl rings and the naphthalene ring are 81.87 (6) and 83.55 (6)°, respectively.

The torsion angles between the carbonyl groups and the naphthalene ring are 113.52 (15)° [C2\C1\C11\O1] and 102.95 (16)° [C8\C9\C18\O2], furthermore those between the carbonyl groups and 3,5-dimethylphenyl groups are 153.91 (13)° [O1\C11\C12\C13] and 164.07 (13)° [O2\C18\C19\C24].

In the crystal structure, the molecular packing of the title compound is stabilized mainly by van der Waals interactions. In addition, the crystal packing is stabilized by three different C—H···O interactions: 1) C7—H7···O1^i^ (Fig. 2 and Table 1). This interaction is directed along the *b* axis. 2) C25—H25*b*···O2^ii^ (Fig. 3 and Table 1). This interaction is directed along the *c* axis. 3) C26—H26*a*···O1^i^ (Fig. 2 and Table 1). This interaction is directed along the *b* axis.

### Experimental

3,5-dimethylbenzoyl chloride (1.50 mmol, 253 mg), titanium chloride (1.50 mmol, 285 mg) and methylene chloride (1.25 ml) were placed into a 10 ml flask, followed by stirring at room temperature. To the reaction mixture thus obtained, 2,7-dimethoxynaphthalene (0.50 mmol, 94.1 mg) was added. The reaction mixture was poured into ice-cold water (30 ml) after it had been stirred for 6 h at room temperature. The aqueous layer was extracted with CHCl_3_ (10 ml × 3). The combined extracts were washed with 2 *M* aqueous NaOH followed by washing with brine. The extracts thus obtained were dried over anhydrous MgSO_4_. The solvent was removed under reduced pressure to give a cake. The crude product was purified by recrystallization from hexane and CHCl_3_ (yield 62%). Colourless platelet single crystals suitable for X-ray diffraction were obtained (the average size: 0.8 × 0.4 × 0.1 mm) by repeated crystallization from hexane/CHCl_3_ mixtures (4:1 *v*/*v*).

^1^H NMR δ (300 MHz, CDCl_3_); 2.24 (12*H*, s), 3.69 (6*H*, s), 7.05 (2*H*, s), 7.21 (2*H*, d, *J* = 9.0 Hz), 7.26 (4*H*, s), 7.95 (2*H*, d, *J* = 9.3 Hz) p.p.m..

^13^C NMR δ (75 MHz, CDCl_3_); 21.19, 56.53, 111.40, 121.94, 124.53, 125.54, 126.99, 131.86, 134.52, 137.19, 138.56, 156.26, 196.94 p.p.m..

IR (KBr); 1656 (C=O), 1610, 1511, 1459 (Ar, naphthalene), 1267 (=C—O—C) cm^-1^.

High-resolution mass spectra (*m/z*); [*M* + Na]^+^ Calcd for C_30_H_28_O_4_Na, 475.1885; found, 475.1851.

m.p. = 576–580 K.

### Refinement

All the H atoms were found in the difference electron density map and were subsequently refined in the riding atom approximation, with C—H = 0.95 (aryl) and 0.98 (methyl) Å, and with *U*_iso_(H) = 1.2*U*_eq_(C_aryl_) and *U*_iso_(H) = 1.5*U*_eq_(C_methyl_). The methyl H atoms C29 are less clear, indicating possible disorder over 4 positions that has not been described in the published model.

### Crystal data

**Table:**

C_30_H_28_O_4_	*F*(000) = 960
*M**_r_* = 452.52	*D*_x_ = 1.260 Mg m^−^^3^
Monoclinic, *P*2_1_/*c*	Melting point = 576–580 K
Hall symbol: -P 2ybc	Cu *K*α radiation, λ = 1.54187 Å
*a* = 19.4659 (3) Å	Cell parameters from 38875 reflections
*b* = 8.27808 (10) Å	θ = 3.0–68.2°
*c* = 15.8244 (2) Å	µ = 0.66 mm^−^^1^
β = 110.69°	*T* = 193 K
*V* = 2385.46 (6) Å^3^	Platelet, colorless
*Z* = 4	0.50 × 0.20 × 0.10 mm

### Data collection

**Table:**

Rigaku R-AXIS RAPID diffractometer	4360 independent reflections
Radiation source: rotating anode	3884 reflections with *I* > 2σ(*I*)
Graphite monochromator	*R*_int_ = 0.032
Detector resolution: 10.000 pixels mm^-1^	θ_max_ = 68.2°, θ_min_ = 4.9°
ω scans	*h* = −23→23
Absorption correction: numerical (*NUMABS*; Higashi, 1999)	*k* = −9→9
*T*_min_ = 0.734, *T*_max_ = 0.937	*l* = −19→19
43008 measured reflections	

### Refinement

**Table:**

Refinement on *F*^2^	Secondary atom site location: difference Fourier map
Least-squares matrix: full	Hydrogen site location: difference Fourier map
*R*[*F*^2^ > 2σ(*F*^2^)] = 0.038	H-atom parameters constrained
*wR*(*F*^2^) = 0.118	*w* = 1/[σ^2^(*F*_o_^2^) + (0.0691*P*)^2^ + 0.4917*P*] where *P* = (*F*_o_^2^ + 2*F*_c_^2^)/3
*S* = 1.08	(Δ/σ)_max_ < 0.001
4360 reflections	Δρ_max_ = 0.22 e Å^−^^3^
314 parameters	Δρ_min_ = −0.16 e Å^−^^3^
0 restraints	Extinction correction: *SHELXL97* (Sheldrick, 2008), Fc^*^=kFc[1+0.001xFc^2^λ^3^/sin(2θ)]^-1/4^
106 constraints	Extinction coefficient: 0.0036 (3)
Primary atom site location: structure-invariant direct methods	

### Special details

**Table:**

Geometry. All e.s.d.'s (except the e.s.d. in the dihedral angle between two l.s. planes) are estimated using the full covariance matrix. The cell e.s.d.'s are taken into account individually in the estimation of e.s.d.'s in distances, angles and torsion angles; correlations between e.s.d.'s in cell parameters are only used when they are defined by crystal symmetry. An approximate (isotropic) treatment of cell e.s.d.'s is used for estimating e.s.d.'s involving l.s. planes.
Refinement. Refinement of *F*^2^ against ALL reflections. The weighted *R*-factor *wR* and goodness of fit *S* are based on *F*^2^, conventional *R*-factors *R* are based on *F*, with *F* set to zero for negative *F*^2^. The threshold expression of *F*^2^ > σ(*F*^2^) is used only for calculating *R*-factors(gt) *etc*. and is not relevant to the choice of reflections for refinement. *R*-factors based on *F*^2^ are statistically about twice as large as those based on *F*, and *R*- factors based on ALL data will be even larger.

### Fractional atomic coordinates and isotropic or equivalent isotropic displacement parameters (Å^2^)

**Table:**

	*x*	*y*	*z*	*U*_iso_*/*U*_eq_	
O1	0.22734 (5)	0.71254 (11)	0.66111 (6)	0.0394 (2)	
O2	0.28061 (5)	0.99466 (12)	0.81457 (6)	0.0453 (3)	
O3	0.35203 (6)	0.73717 (13)	0.54561 (7)	0.0537 (3)	
O4	0.15557 (6)	1.27961 (12)	0.73319 (8)	0.0523 (3)	
C1	0.28767 (6)	0.91046 (15)	0.60689 (8)	0.0338 (3)	
C2	0.31864 (7)	0.88289 (17)	0.54171 (9)	0.0410 (3)	
C3	0.31234 (8)	0.9973 (2)	0.47309 (9)	0.0497 (4)	
H3	0.3334	0.9761	0.4284	0.060*	
C4	0.27599 (8)	1.1377 (2)	0.47163 (9)	0.0493 (4)	
H4	0.2717	1.2140	0.4252	0.059*	
C5	0.24450 (7)	1.17353 (17)	0.53693 (9)	0.0410 (3)	
C6	0.20817 (8)	1.32174 (18)	0.53500 (10)	0.0482 (4)	
H6	0.2054	1.3975	0.4888	0.058*	
C7	0.17710 (8)	1.35995 (17)	0.59644 (10)	0.0473 (4)	
H7	0.1527	1.4604	0.5932	0.057*	
C8	0.18160 (7)	1.24812 (16)	0.66535 (10)	0.0412 (3)	
C9	0.21571 (6)	1.09969 (15)	0.67024 (9)	0.0343 (3)	
C10	0.24920 (6)	1.05758 (15)	0.60622 (8)	0.0340 (3)	
C11	0.28661 (7)	0.77005 (14)	0.66698 (8)	0.0319 (3)	
C12	0.35610 (7)	0.70057 (15)	0.73078 (8)	0.0320 (3)	
C13	0.41949 (7)	0.79286 (16)	0.76626 (8)	0.0360 (3)	
H13	0.4196	0.9018	0.7475	0.043*	
C14	0.48268 (7)	0.72703 (18)	0.82903 (9)	0.0407 (3)	
C15	0.48139 (7)	0.56487 (18)	0.85289 (9)	0.0425 (3)	
H15	0.5246	0.5181	0.8947	0.051*	
C16	0.41900 (7)	0.46975 (17)	0.81758 (9)	0.0399 (3)	
C17	0.35596 (7)	0.53999 (16)	0.75728 (9)	0.0356 (3)	
H17	0.3122	0.4780	0.7338	0.043*	
C18	0.22180 (7)	1.00106 (14)	0.75278 (8)	0.0336 (3)	
C19	0.15522 (6)	0.91939 (14)	0.75809 (8)	0.0327 (3)	
C20	0.15643 (7)	0.86210 (15)	0.84140 (9)	0.0367 (3)	
H20	0.1990	0.8777	0.8936	0.044*	
C21	0.09618 (8)	0.78263 (16)	0.84899 (9)	0.0411 (3)	
C22	0.03586 (7)	0.75515 (16)	0.77074 (10)	0.0412 (3)	
H22	−0.0053	0.6990	0.7751	0.049*	
C23	0.03382 (7)	0.80700 (16)	0.68642 (9)	0.0392 (3)	
C24	0.09358 (7)	0.89291 (15)	0.68109 (9)	0.0352 (3)	
H24	0.0923	0.9338	0.6245	0.042*	
C25	0.38607 (10)	0.7042 (2)	0.48124 (11)	0.0560 (4)	
H25A	0.4240	0.7854	0.4866	0.084*	
H25B	0.3491	0.7076	0.4202	0.084*	
H25C	0.4086	0.5967	0.4926	0.084*	
C26	0.12856 (10)	1.43782 (19)	0.73930 (15)	0.0627 (5)	
H26A	0.1665	1.5177	0.7422	0.094*	
H26B	0.1159	1.4456	0.7939	0.094*	
H26C	0.0847	1.4587	0.6860	0.094*	
C27	0.55064 (9)	0.8269 (2)	0.87241 (12)	0.0620 (5)	
H27A	0.5428	0.9359	0.8466	0.093*	
H27B	0.5922	0.7766	0.8612	0.093*	
H27C	0.5612	0.8332	0.9376	0.093*	
C28	0.42004 (9)	0.29395 (19)	0.84383 (13)	0.0567 (4)	
H28A	0.4598	0.2381	0.8313	0.085*	
H28B	0.3730	0.2438	0.8088	0.085*	
H28C	0.4281	0.2860	0.9084	0.085*	
C29	0.09670 (11)	0.7238 (2)	0.93949 (11)	0.0632 (5)	
H29A	0.1082	0.8141	0.9823	0.095*	
H29B	0.1340	0.6393	0.9621	0.095*	
H29C	0.0483	0.6798	0.9328	0.095*	
C30	−0.03058 (8)	0.7652 (2)	0.60251 (11)	0.0543 (4)	
H30A	−0.0745	0.7500	0.6183	0.081*	
H30B	−0.0200	0.6652	0.5762	0.081*	
H30C	−0.0390	0.8531	0.5585	0.081*	

### Atomic displacement parameters (Å^2^)

**Table:**

	*U*^11^	*U*^22^	*U*^33^	*U*^12^	*U*^13^	*U*^23^
O1	0.0349 (5)	0.0337 (5)	0.0511 (5)	−0.0012 (4)	0.0170 (4)	0.0029 (4)
O2	0.0366 (5)	0.0497 (6)	0.0424 (5)	−0.0041 (4)	0.0050 (4)	0.0061 (4)
O3	0.0734 (7)	0.0547 (6)	0.0447 (6)	0.0092 (5)	0.0353 (5)	0.0027 (5)
O4	0.0599 (6)	0.0322 (5)	0.0717 (7)	0.0075 (4)	0.0315 (6)	0.0012 (5)
C1	0.0322 (6)	0.0381 (7)	0.0284 (6)	−0.0039 (5)	0.0074 (5)	0.0023 (5)
C2	0.0418 (7)	0.0465 (8)	0.0344 (7)	−0.0034 (6)	0.0130 (6)	0.0004 (6)
C3	0.0536 (8)	0.0652 (10)	0.0326 (7)	−0.0090 (7)	0.0181 (6)	0.0043 (6)
C4	0.0515 (8)	0.0559 (9)	0.0355 (7)	−0.0080 (7)	0.0090 (6)	0.0151 (6)
C5	0.0367 (6)	0.0427 (7)	0.0356 (7)	−0.0083 (6)	0.0027 (5)	0.0091 (6)
C6	0.0469 (8)	0.0382 (7)	0.0458 (8)	−0.0065 (6)	−0.0007 (6)	0.0152 (6)
C7	0.0423 (7)	0.0307 (7)	0.0579 (9)	−0.0005 (6)	0.0041 (6)	0.0078 (6)
C8	0.0343 (6)	0.0312 (7)	0.0524 (8)	−0.0025 (5)	0.0083 (6)	0.0010 (6)
C9	0.0292 (6)	0.0302 (6)	0.0392 (7)	−0.0033 (5)	0.0068 (5)	0.0024 (5)
C10	0.0300 (6)	0.0342 (6)	0.0320 (6)	−0.0060 (5)	0.0035 (5)	0.0038 (5)
C11	0.0349 (6)	0.0304 (6)	0.0326 (6)	−0.0009 (5)	0.0145 (5)	−0.0027 (5)
C12	0.0350 (6)	0.0350 (6)	0.0299 (6)	0.0021 (5)	0.0166 (5)	0.0010 (5)
C13	0.0396 (7)	0.0382 (7)	0.0330 (6)	−0.0018 (5)	0.0162 (5)	0.0046 (5)
C14	0.0371 (7)	0.0497 (8)	0.0364 (7)	−0.0026 (6)	0.0144 (6)	0.0053 (6)
C15	0.0362 (7)	0.0514 (8)	0.0408 (7)	0.0080 (6)	0.0149 (6)	0.0099 (6)
C16	0.0412 (7)	0.0383 (7)	0.0448 (7)	0.0071 (5)	0.0209 (6)	0.0062 (6)
C17	0.0366 (6)	0.0354 (7)	0.0386 (7)	0.0014 (5)	0.0182 (5)	0.0006 (5)
C18	0.0352 (6)	0.0281 (6)	0.0366 (6)	0.0010 (5)	0.0113 (5)	−0.0025 (5)
C19	0.0352 (6)	0.0275 (6)	0.0363 (6)	0.0029 (5)	0.0137 (5)	−0.0028 (5)
C20	0.0419 (7)	0.0328 (7)	0.0354 (6)	0.0007 (5)	0.0135 (5)	−0.0046 (5)
C21	0.0493 (8)	0.0362 (7)	0.0436 (7)	0.0007 (6)	0.0235 (6)	−0.0021 (6)
C22	0.0386 (7)	0.0366 (7)	0.0549 (8)	−0.0009 (5)	0.0246 (6)	−0.0026 (6)
C23	0.0332 (6)	0.0372 (7)	0.0466 (7)	0.0021 (5)	0.0131 (5)	−0.0052 (6)
C24	0.0352 (6)	0.0342 (7)	0.0367 (6)	0.0033 (5)	0.0134 (5)	−0.0003 (5)
C25	0.0661 (10)	0.0657 (10)	0.0449 (8)	−0.0039 (8)	0.0305 (8)	−0.0111 (7)
C26	0.0634 (10)	0.0313 (7)	0.1061 (14)	0.0047 (7)	0.0459 (10)	0.0001 (8)
C27	0.0479 (8)	0.0714 (11)	0.0542 (9)	−0.0144 (8)	0.0024 (7)	0.0161 (8)
C28	0.0537 (9)	0.0416 (8)	0.0746 (11)	0.0102 (7)	0.0226 (8)	0.0151 (7)
C29	0.0749 (11)	0.0720 (11)	0.0509 (9)	−0.0124 (9)	0.0326 (8)	0.0033 (8)
C30	0.0383 (7)	0.0636 (10)	0.0552 (9)	−0.0076 (7)	0.0094 (7)	−0.0055 (7)

### Geometric parameters (Å, º)

**Table:**

O1—C11	1.2208 (15)	C16—C28	1.512 (2)
O2—C18	1.2158 (15)	C17—H17	0.9500
O3—C2	1.3613 (18)	C18—C19	1.4903 (17)
O3—C25	1.4242 (17)	C19—C24	1.3926 (17)
O4—C8	1.3644 (18)	C19—C20	1.3935 (18)
O4—C26	1.4272 (18)	C20—C21	1.3865 (19)
C1—C2	1.3853 (18)	C20—H20	0.9500
C1—C10	1.4278 (18)	C21—C22	1.392 (2)
C1—C11	1.5065 (17)	C21—C29	1.509 (2)
C2—C3	1.413 (2)	C22—C23	1.389 (2)
C3—C4	1.356 (2)	C22—H22	0.9500
C3—H3	0.9500	C23—C24	1.3912 (19)
C4—C5	1.407 (2)	C23—C30	1.5094 (19)
C4—H4	0.9500	C24—H24	0.9500
C5—C6	1.411 (2)	C25—H25A	0.9800
C5—C10	1.4356 (18)	C25—H25B	0.9800
C6—C7	1.351 (2)	C25—H25C	0.9800
C6—H6	0.9500	C26—H26A	0.9800
C7—C8	1.409 (2)	C26—H26B	0.9800
C7—H7	0.9500	C26—H26C	0.9800
C8—C9	1.3857 (18)	C27—H27A	0.9800
C9—C10	1.4282 (18)	C27—H27B	0.9800
C9—C18	1.5090 (18)	C27—H27C	0.9800
C11—C12	1.4882 (17)	C28—H28A	0.9800
C12—C13	1.3899 (18)	C28—H28B	0.9800
C12—C17	1.3942 (18)	C28—H28C	0.9800
C13—C14	1.3904 (18)	C29—H29A	0.9800
C13—H13	0.9500	C29—H29B	0.9800
C14—C15	1.397 (2)	C29—H29C	0.9800
C14—C27	1.504 (2)	C30—H30A	0.9800
C15—C16	1.388 (2)	C30—H30B	0.9800
C15—H15	0.9500	C30—H30C	0.9800
C16—C17	1.3881 (18)		
			
C2—O3—C25	118.22 (12)	C19—C18—C9	119.36 (10)
C8—O4—C26	118.47 (12)	C24—C19—C20	119.70 (12)
C2—C1—C10	120.01 (11)	C24—C19—C18	121.32 (11)
C2—C1—C11	116.72 (11)	C20—C19—C18	118.91 (11)
C10—C1—C11	122.59 (11)	C21—C20—C19	120.76 (12)
O3—C2—C1	116.02 (12)	C21—C20—H20	119.6
O3—C2—C3	122.64 (13)	C19—C20—H20	119.6
C1—C2—C3	121.28 (13)	C20—C21—C22	118.30 (12)
C4—C3—C2	119.38 (13)	C20—C21—C29	120.87 (14)
C4—C3—H3	120.3	C22—C21—C29	120.81 (13)
C2—C3—H3	120.3	C23—C22—C21	122.22 (12)
C3—C4—C5	121.87 (13)	C23—C22—H22	118.9
C3—C4—H4	119.1	C21—C22—H22	118.9
C5—C4—H4	119.1	C22—C23—C24	118.40 (12)
C4—C5—C6	120.83 (13)	C22—C23—C30	120.47 (13)
C4—C5—C10	119.56 (13)	C24—C23—C30	121.08 (13)
C6—C5—C10	119.61 (13)	C23—C24—C19	120.52 (12)
C7—C6—C5	122.42 (13)	C23—C24—H24	119.7
C7—C6—H6	118.8	C19—C24—H24	119.7
C5—C6—H6	118.8	O3—C25—H25A	109.5
C6—C7—C8	118.79 (13)	O3—C25—H25B	109.5
C6—C7—H7	120.6	H25A—C25—H25B	109.5
C8—C7—H7	120.6	O3—C25—H25C	109.5
O4—C8—C9	115.42 (12)	H25A—C25—H25C	109.5
O4—C8—C7	122.99 (13)	H25B—C25—H25C	109.5
C9—C8—C7	121.55 (14)	O4—C26—H26A	109.5
C8—C9—C10	120.43 (12)	O4—C26—H26B	109.5
C8—C9—C18	114.69 (12)	H26A—C26—H26B	109.5
C10—C9—C18	124.47 (11)	O4—C26—H26C	109.5
C1—C10—C9	124.93 (11)	H26A—C26—H26C	109.5
C1—C10—C5	117.89 (12)	H26B—C26—H26C	109.5
C9—C10—C5	117.18 (12)	C14—C27—H27A	109.5
O1—C11—C12	120.59 (11)	C14—C27—H27B	109.5
O1—C11—C1	118.44 (11)	H27A—C27—H27B	109.5
C12—C11—C1	120.96 (10)	C14—C27—H27C	109.5
C13—C12—C17	119.89 (12)	H27A—C27—H27C	109.5
C13—C12—C11	121.74 (11)	H27B—C27—H27C	109.5
C17—C12—C11	118.34 (11)	C16—C28—H28A	109.5
C12—C13—C14	120.54 (12)	C16—C28—H28B	109.5
C12—C13—H13	119.7	H28A—C28—H28B	109.5
C14—C13—H13	119.7	C16—C28—H28C	109.5
C13—C14—C15	118.28 (12)	H28A—C28—H28C	109.5
C13—C14—C27	121.61 (13)	H28B—C28—H28C	109.5
C15—C14—C27	120.10 (13)	C21—C29—H29A	109.5
C16—C15—C14	122.20 (12)	C21—C29—H29B	109.5
C16—C15—H15	118.9	H29A—C29—H29B	109.5
C14—C15—H15	118.9	C21—C29—H29C	109.5
C17—C16—C15	118.31 (12)	H29A—C29—H29C	109.5
C17—C16—C28	120.99 (13)	H29B—C29—H29C	109.5
C15—C16—C28	120.70 (13)	C23—C30—H30A	109.5
C16—C17—C12	120.73 (12)	C23—C30—H30B	109.5
C16—C17—H17	119.6	H30A—C30—H30B	109.5
C12—C17—H17	119.6	C23—C30—H30C	109.5
O2—C18—C19	121.69 (12)	H30A—C30—H30C	109.5
O2—C18—C9	118.91 (11)	H30B—C30—H30C	109.5
			
C25—O3—C2—C1	−178.72 (12)	C10—C1—C11—C12	−124.21 (12)
C25—O3—C2—C3	4.3 (2)	O1—C11—C12—C13	−153.92 (12)
C10—C1—C2—O3	−177.78 (11)	C1—C11—C12—C13	27.31 (17)
C11—C1—C2—O3	−7.02 (17)	O1—C11—C12—C17	23.92 (17)
C10—C1—C2—C3	−0.73 (19)	C1—C11—C12—C17	−154.85 (11)
C11—C1—C2—C3	170.02 (12)	C17—C12—C13—C14	−0.87 (18)
O3—C2—C3—C4	177.62 (13)	C11—C12—C13—C14	176.94 (11)
C1—C2—C3—C4	0.8 (2)	C12—C13—C14—C15	2.17 (19)
C2—C3—C4—C5	0.3 (2)	C12—C13—C14—C27	−176.47 (14)
C3—C4—C5—C6	178.77 (13)	C13—C14—C15—C16	−1.4 (2)
C3—C4—C5—C10	−1.4 (2)	C27—C14—C15—C16	177.23 (14)
C4—C5—C6—C7	179.79 (13)	C14—C15—C16—C17	−0.6 (2)
C10—C5—C6—C7	0.0 (2)	C14—C15—C16—C28	178.99 (14)
C5—C6—C7—C8	0.5 (2)	C15—C16—C17—C12	1.98 (19)
C26—O4—C8—C9	171.87 (13)	C28—C16—C17—C12	−177.64 (13)
C26—O4—C8—C7	−5.8 (2)	C13—C12—C17—C16	−1.26 (18)
C6—C7—C8—O4	176.22 (13)	C11—C12—C17—C16	−179.14 (11)
C6—C7—C8—C9	−1.3 (2)	C8—C9—C18—O2	−102.93 (14)
O4—C8—C9—C10	−176.03 (11)	C10—C9—C18—O2	69.75 (17)
C7—C8—C9—C10	1.64 (19)	C8—C9—C18—C19	74.74 (14)
O4—C8—C9—C18	−3.02 (16)	C10—C9—C18—C19	−112.58 (13)
C7—C8—C9—C18	174.65 (12)	O2—C18—C19—C24	−164.08 (12)
C2—C1—C10—C9	−179.42 (11)	C9—C18—C19—C24	18.31 (17)
C11—C1—C10—C9	10.39 (18)	O2—C18—C19—C20	12.90 (18)
C2—C1—C10—C5	−0.37 (17)	C9—C18—C19—C20	−164.70 (11)
C11—C1—C10—C5	−170.56 (11)	C24—C19—C20—C21	−1.70 (19)
C8—C9—C10—C1	177.91 (11)	C18—C19—C20—C21	−178.73 (11)
C18—C9—C10—C1	5.62 (19)	C19—C20—C21—C22	2.87 (19)
C8—C9—C10—C5	−1.15 (17)	C19—C20—C21—C29	−178.60 (14)
C18—C9—C10—C5	−173.44 (11)	C20—C21—C22—C23	−1.1 (2)
C4—C5—C10—C1	1.42 (18)	C29—C21—C22—C23	−179.68 (14)
C6—C5—C10—C1	−178.78 (11)	C21—C22—C23—C24	−1.7 (2)
C4—C5—C10—C9	−179.46 (11)	C21—C22—C23—C30	175.97 (13)
C6—C5—C10—C9	0.35 (17)	C22—C23—C24—C19	2.93 (19)
C2—C1—C11—O1	−113.50 (13)	C30—C23—C24—C19	−174.75 (12)
C10—C1—C11—O1	56.99 (17)	C20—C19—C24—C23	−1.27 (18)
C2—C1—C11—C12	65.29 (15)	C18—C19—C24—C23	175.69 (11)

### Hydrogen-bond geometry (Å, º)

**Table:**

*D*—H···*A*	*D*—H	H···*A*	*D*···*A*	*D*—H···*A*
C7—H7···O1^i^	0.95	2.55	3.1332 (17)	120
C25—H25*B*···O2^ii^	0.98	2.41	3.170 (2)	134
C26—H26*A*···O1^i^	0.98	2.59	3.475 (2)	150

Symmetry codes: (i) *x*, *y*+1, *z*; (ii) *x*, −*y*+3/2, *z*−1/2.
